# Auricular Acupuncture and Vagal Regulation

**DOI:** 10.1155/2012/786839

**Published:** 2012-11-27

**Authors:** Wei He, Xiaoyu Wang, Hong Shi, Hongyan Shang, Liang Li, Xianghong Jing, Bing Zhu

**Affiliations:** Institute of Acupuncture and Moxibustion, China Academy of Chinese Medical Sciences, Beijing 100700, China

## Abstract

Auricular acupuncture has been utilized in the treatment of diseases for thousands of years. Dr. Paul Nogier firstly originated the concept of an inverted fetus map on the external ear. In the present study, the relationship between the auricular acupuncture and the vagal regulation has been reviewed. It has been shown that auricular acupuncture plays a role in vagal activity of autonomic functions of cardiovascular, respiratory, and gastrointestinal systems. Mechanism studies suggested that afferent projections from especially the auricular branch of the vagus nerve (ABVN) to the nucleus of the solitary tract (NTS) form the anatomical basis for the vagal regulation of auricular acupuncture. Therefore, we proposed the “auriculovagal afferent pathway” (AVAP): both the autonomic and the central nervous system could be modified by auricular vagal stimulation via projections from the ABVN to the NTS. Auricular acupuncture is also proposed to prevent neurodegenerative diseases via vagal regulation. There is a controversy on the specificity and the efficacy of auricular acupoints for treating diseases. More clinical RCT trials on auricular acupuncture and experimental studies on the mechanism of auricular acupuncture should be further investigated.

## 1. The History of Auricular Acupuncture

Acupuncture is a part of traditional Chinese medicine (TCM). It has been accepted in China and has been used as one of the alternative and complementary treatments in western countries. Auricular acupuncture has been also used in the treatment of diseases for thousands of years. In the classic TCM text of Huang Di Nei Jing, which was compiled in around 500 B.C, the correlation between the auricle and the body had been described; all six Yang meridians were directly connected to the auricle, whereas the six Yin meridians were indirectly connected to the ear by their corresponding yang meridian, respectively [1]. In Hippocrates' time, around 450 BC, bleeding points on the posterior (mastoid) surface of the ear were used to facilitate ejaculation, reduce impotency problem, and treat leg pain [2]. It was also reported that the auricle was associated with emotion [2]. During Renaissance sporadic trading between China and Europe made it possible to introduce needles, moxa, and cauterization of the external ear or cutting the veins behind the ears for relieving diseases in Europe [3]. In 1957, Dr. Paul Nogier, a physician in France, firstly originated the concept of an inverted fetus map on the external ear [2]. He proposed the concept after visiting a folk doctor, who cauterized the very small auricular area “sciatic point” of the patients for the treatment of sciatica. The folk doctor learned this technique from a Chinese who resided in Marseilles [3]. 

Nogier presented his discovery in several congresses and published it in an international circulation journal, which eventually led to the widespread acceptance of his approach. With some exceptions, the Chinese charts were very similar to Nogier's originals [4]. 

## 2. Auricular Acupuncture for Vagal Regulation

The autonomic nervous system (ANS), which plays a crucial role in the maintenance of homeostasis, is mainly composed of two anatomically and functionally distinct divisions: the sympathetic system and the parasympathetic system. In terms of the influence of the parasympathetic system, the physiological significance of the vagus nerve is clearly illustrated by its widespread distribution [5]. It controls the activity of the cardiovascular, respiratory, and gastrointestinal systems and has effects on smooth muscles, blood vessels, sweat glands, and the endocrine system. Numerous investigations showed that vagal tone was elicited by auricular acupuncture or auricular acupressure [6–8]. It is described as a reflexive treatment of physical, emotional, and neurological dysfunctions via specific zones on the ear where these dysfunctions are reflected [9]. 

### 2.1. Cardiovascular Regulation

Cardiac vagal postganglionic fiber endings release acetylcholine, which are bound with cholinergic M receptors on the myocardial cell membrane or vascular smooth muscle. Activation of the vagus nerve typically leads to a reduction in heart rate and blood pressure. Cardiovascular vagal regulations by auricular acupuncture have been investigated in clinical trials and animal experiments [6, 10–18]. In elite basketball athletes, the value of heart rate decreased at 30th and 60th minutes postexercise in auricular acupuncture group compared with that in normal control group [6]. In fourteen healthy men, auricular electrical acupuncture stimulation was found to have a positive effect on respiratory sinus arrhythmia adjusted for tidal volume, which indicated an increase in vagal activity [10]. The systolic pressure and diastolic pressure in 20 cases of hypertension rabbits were decreased by ear electro-acupuncture inserting at the “Er Jian” (HX_6.7*i*_) point [11]. Acupuncture at “shenmen” (TF4) slowed down the heart rate and activated the parasympathetic nerves [12].

Several investigations had focused on the relationship between auricular acupoint “Heart” (CO_15_) and cardiovascular regulation. In healthy volunteers, a significant decrease in heart rate and a significant increase in heart rate variability after manual ear acupressure at auricular acupoint CO_15_ have been shown [13]. In anesthetized Sprague Dawley rats, acupuncture at auricular point “Heart” showed a more significant inhibitory effect on arterial pressure and heart rate than acupuncture at acupoints Zusanli (ST36) and Neiguan (PC6) [14]. Decrease in blood pressure and a small bradycardia had been induced by auricular acupuncture at different points in rats [8]. A significant increase in total heart rate variability was found after auricular acupuncture at the ear point CO_15_ [15]. In addition, mean blood flow velocity of the ophthalmic artery was significantly increased during needling vision-related acupoints of auricular acupuncture, which may be induced by parasympathetic tone [16]. In 30 cases of vascular hypertensive patients, it was found that acupuncture at acupoint CO_15_ produced marked short-term and long-term depressor effect as well as evident immediate effects on cardiac functional activities in grade II and grade III hypertension and marked effects on angiotensin II in grade III hypertension [17]. 

After receiving 4-week-treatment of auricular acupuncture therapy, a greater percentage change in Pittsburgh sleep quality index was moderately correlated with both a lower percentage change in high frequency power of heart rate variability (HRV) and a greater percentage change in normalized low frequency power of HRV, thus, it suggested that auricular acupuncture intervention led to more cardiac parasympathetic and less cardiac sympathetic activities, which contributed to the improvement of postmenopausal insomnia [18]. 

### 2.2. Respiratory Regulation

In a controlled single-blind study, a significant decrease in the olfactory recognition threshold by auricular acupuncture at the auricular “Lung” point was found in 23 healthy volunteers [7]. Bilateral stimulation of auricular acupoint TF_4_ combined with other acupoints of Daimai (GB26), ST36, and Sanyinjiao (SP6) resulted in a net increase in vital capacity during the period of acupuncture analgesia which lasted for 3 to 4 hours after stimulation [19]. In fourteen healthy men, auricular electrical accupuncture stimulation was found to have a positive effect on respiratory sinus arrhythmia adjusted for tidal volume, which indicated an increase in vagal activity [10]. 

### 2.3. Gastrointestinal Regulation

Increase in intragastric pressure has been induced by auricular acupuncture in rats [8]. By comparison of the width of corpus and antrum of the stomach, as well as duodenum before and after the application of auricular acupuncture in 60 patients, the results showed that the effects of auricular acupuncture and usual drugs on the motility and tone of gastrointestinal tract were equal [20]. In order to relieve the abdominal distension and other discomforts due to gastrointestinal dysfunction after abdominal operations, the patients were treated by auricular-plaster therapy plus acupuncture at ST36. The results indicated that auricular-plaster therapy plus acupuncture at ST36 may promote postoperative recovery of the intestinal function [21].

## 3. Mechanisms of Auricular Acupuncture for Vagal Regulation 

### 3.1. The Nerve Supply of the Auricle

**Figure 1:**
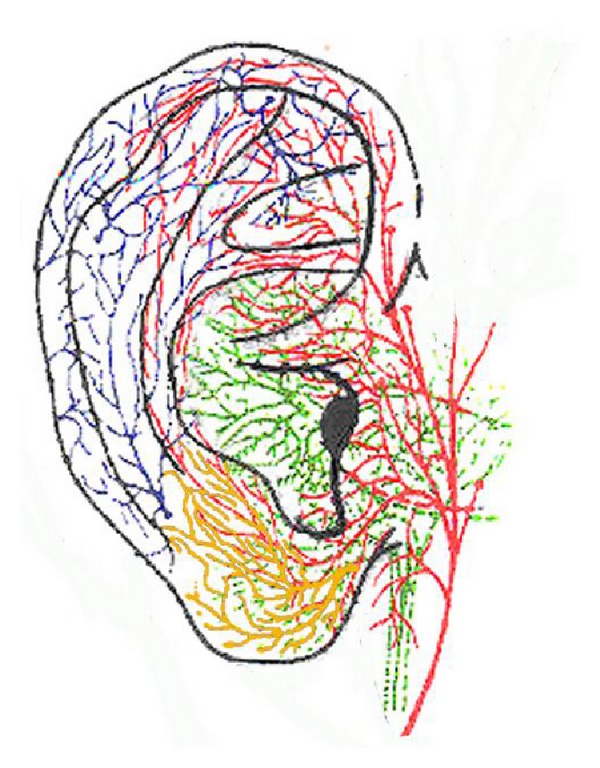
The innervations of the external auricle. The innervations of the auricular branch of the vagus nerve are marked by green color. The innervations of the auriculotemporal nerve are marked by red color. The innervations of the lesser occipital nerve are marked by blue color. The innervations of the greater auricular nerve are marked by yellow color.

The auricle is innervated by cranial nerves and spinal nerves. Innervations of at least four nerves supply the anterior auricle: the auriculotemporal nerve, the auricular branch of the vagus nerve (ABVN), the lesser occipital nerve, and the greater auricular nerve. The auriculotemporal nerve is a mandibular branch of the trigeminal nerve, which mainly supplies the anterosuperior and anteromedial areas of the external ear. The auricular branch of the vagus nerve, which is the only peripheral branch of the vagus nerve, mainly supplies the auricular concha and most of the area around the auditory meatus. The lesser occipital nerve mainly innervates the skin of the upper and back parts of the auricular. The greater auricular nerve (GAN) from the cervical plexus supplies both surfaces of the lower parts of the auricle. The innervation of the auricle is characterized by a great deal of overlap between multiple nerves [22] (see Figure 1). 

### 3.2. Auriculovagal Relation

**Figure 2:**
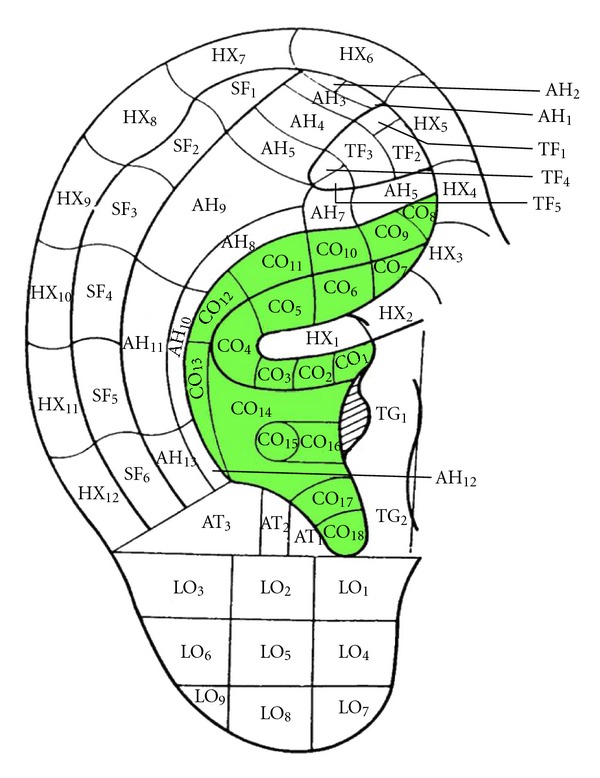
Auricular acupoints treating visceral diseases are mainly located at auricular concha.

Both Chinese and Western researchers have recognized the relationship between the auricle and vagal regulation. Arnold's reflex was first described in 1832 by Friedrich Arnold, professor of anatomy at Heidelberg University in Germany. It is one of the somato-parasympathetic reflexes. Physical stimulation of the external acoustic meatus innervated by the ABVN elicits a cough much like the other cough reflexes induced by vagal tone. There were also clinic reports on vagal tone responses such as cardiac deceleration and even asystole and depressor response, induced by stimulations including cerumen cramming in auditory canal or auricular concha [23, 24]. Engel [25] groups together eight reflexes including gastroauricular phenomenon in man, auricular phenomenon in man, pulmonoauricular phenomenon in man, auriculogenital reflex in cat, auriculouterine reflex in women, oculocardiac reflex in man, Kalchschmidt's reflex in cattle, and coughing attack with heartburn in man. According to the national standards of the location of auricular acupoints [26], auricular acupoints treating visceral diseases are mainly located at auricular concha (see Figure 2). Perhaps the ABVN forms a connection between the auricle and the autonomic regulations. 

### 3.3. Relationship between the ABVN and the Nucleus of the Solitary Tract

The anatomical relationship between the ABVN and the nucleus of the solitary tract (NTS) has been investigated. After applying horseradish peroxidase (HRP) to the central cut end of the ABVN in the cat, some labeled neuronal terminals were seen in the interstitial, dorsal, dorsolateral, and commissural subnuclei of the NTS; some of these terminals may be connected monosynaptically with solitary nucleus neurons which send their axons to visceromotor centers in the brainstem [27]. 

The auricular concha is mainly innervated by the ABVN. The relationship between the acupuncture stimulation at auricular concha and the NTS has also been investigated. In an animal study, acupuncture stimulation at auricular concha induced the hypoglycemic effect by activating the firing activities of the neurons in NTS [28]. It is also found that acupuncture-like stimulation at auricular acupoint CO_15_ activates the cardiac-related neurons in the NTS to evoke cardiovascular inhibition, whereas the inactivation of the NTS with local anesthetics decreased the cardiovascular inhibitory responses evoked by auricular acupuncture [14]. 

Recently, it is suggested to assess the function of the vagus nerve through transcutaneous electric stimulation of the ABVN innervating parts of the ear. The 8 mA stimulation was performed at five different electrode positions at the subject's right ear. A clear, reproducible vagus sensory evoked potential (VSEP) was recorded after stimulation at the inner side of the tragus of the right ear, instead of the other stimulation positions at the lobulus auriculae, the scapha, thecus antihelices superior, and the top of the helix. It is considered that cutaneous stimuli of this region are transported via the auricular nerve to the jugular ganglion and from there with the vagus nerve into the medulla oblongata and to the NTS [29]. Although other regions of the auricle might be innervated by a small amount of innervation of the ABVN, the inner side of the tragus is a large amount of innervation the ABVN to mediate the VSEP.

### 3.4. Extensive Connections between the NTS with Visceral Organs and Other Brain Structures

The NTS in the brainstem carries and receives visceral primary afferent signals from a variety of visceral regions and organs. Neurons that synapse in the NTS participate into the autonomic reflexes, with a result to regulate the autonomic function. Outputs that go from the NTS are transferred to a large number of other regions of the brain including the paraventricular nucleus of the hypothalamus and the central nucleus of the amygdala as well as to other nuclei in the brainstem (such as the parabrachial area and other visceral motor or respiratory networks). Perhaps, extensive connections between the NTS with visceral organs and other brain structures [30] may elucidate the mechanism of auricular acupuncture. 

**Figure 3:**
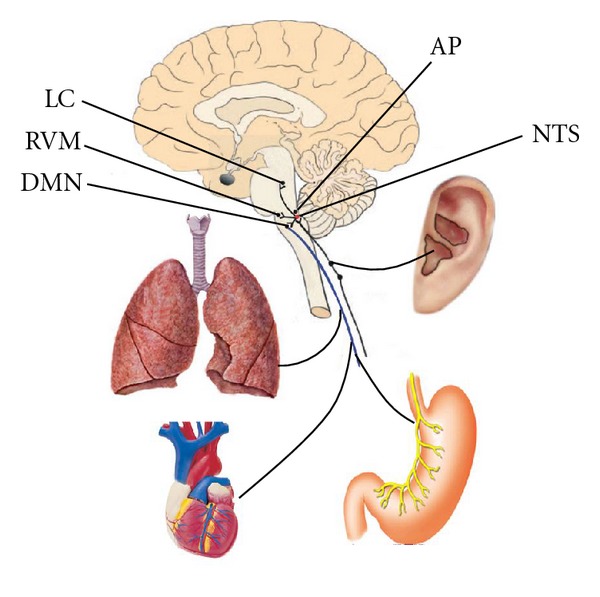
“Auriculovagal afferent pathway” (AVAP): both the autonomic and the central nervous system could be modified by auricular vagal stimulation via projections from the ABVN at the auricular concha to the NTS (see Figure 3). NTS: nucleus of the solitary tract; DMN: dorsal motor nucleus of the vagus; AP: area postrema; RVM: rostral ventrolateral medulla; LC: locus coeruleus.

Therefore, we proposed the “auriculovagal afferent pathway” (AVAP); both the autonomic and the central nervous system could be modified by auricular vagal stimulation via projections from the ABVN to the NTS (see Figure 3). 

## 4. Prevention and Treatment of Diseases via Vagal Regulation of Auricular Acupuncture

The nuclei of the vagus nerve in the brainstem have been implicated as one of the earliest regions in the pathophysiological process of both Alzheimer's and Parkinson's diseases. Far-field potentials from brainstem after transcutaneous vagus nerve stimulation at the auricle have been utilized as a noninvasive method in the early diagnosis of neurodegenerative disorders [31–33]. We suggest that further study is needed on whether auricular acupuncture plays a role in the prevention and treatment of these neurodegenerative disorders via activating the vagal nuclei in the brainstem. 

Vagus nerve stimulation has been approved by FDA as an alternative treatment for neuropsychiatric diseases such as epilepsy and depression. In order to avoid the disadvantages of cervical vagus nerve stimulation, less invasive methods including transcutaneous vagus nerve stimulation [34–36] and electrical auricula-vagus stimulation [37] to stimulate vagal afferences have been proposed. In a pilot study, an overall reduction of seizure frequency was observed in five of seven patients after 9 months of electrical stimulation of the ABVN. It is also found that the electrical stimulation of the ABVN is safe and well tolerated [38]. As a complementary method, it is also proposed that auricular acupuncture may suppress epileptic seizures via activating the parasympathetic nervous system [39–41].

## 5. Complications on Auricular Acupuncture

### 5.1. Controversy on Specificity of Auricular Acupoint

Several studies investigated the specificity of auricular acupoints. Parts of the studies agree on the concept that specific areas of the ear are related to specific areas of the body. Acupuncture at CO_15_, but not Stomach (CO_4_), produced depressor effect on vascular hypertension [17, 42]. Specificity of auricular acupoint is also identified by two quantified examinations of the electrical properties [43, 44]. 

There is still disagreement on the specificity of auricular acupoint. Similar patterns of cardiovascular and gastric responses could be evoked by stimulation at different areas of the auricle, which do not support the theory of a highly specific functional map in the ear [8]. Auricular acupuncture appears to be effective for smoking cessation, but the effect may not depend on point location [45]. 

### 5.2. Inconsistent Results on the Study of Auricular Acupuncture

There are inconsistent study results related to the treatment effects of auricular acupuncture, which may be related to trial designing, clinical observation measures, the set of sham acupuncture, and statistical analyses [46–48]. In clinical studies, most studies on the clinical observation of auricular acupuncture were not sufficiently convincing. More RCT evaluations of effect of auricular acupuncture should be performed to obtain objective and consistent results. Besides, there are almost 200 auricular acupoints in each ear that represent all parts of the body and many functional areas. It is not easy to locate the acupoint accurately. Therefore, in a clinical trial, the acupuncture operator should be trained well. In experimental studies, anatomical and morphological studies on auricular acupoints and neuroimaging study such as fMRI on the effect of auricular acupuncture should be encouraged to investigate the mechanism of auricular acupuncture.
